# Reduced cortical gyrification in the posteromedial cortex in unaffected relatives of schizophrenia patients with high genetic loading

**DOI:** 10.1038/s41537-021-00148-1

**Published:** 2021-03-01

**Authors:** Inkyung Park, Minah Kim, Tae Young Lee, Wu Jeong Hwang, Yoo Bin Kwak, Sanghoon Oh, Silvia Kyungjin Lho, Sun-Young Moon, Jun Soo Kwon

**Affiliations:** 1 Department of Brain and Cognitive Sciences, Seoul National University College of Natural Sciences, Seoul, Republic of Korea; 2 Department of Psychiatry, Seoul National University College of Medicine, Seoul, Republic of Korea; 3 Department of Neuropsychiatry, Seoul National University Hospital, Seoul, Republic of Korea; 4 Institute of Human Behavioral Medicine, SNU-MRC, Seoul, Republic of Korea; 5 Department of Neuropsychiatry, Pusan National University Yangsan Hospital, Yangsan, Republic of Korea; 6 Department of Psychiatry, Eulji University Uijeongbu Eulji Medical Center, Gyeonggi, Republic of Korea

**Keywords:** Neuroscience, Biomarkers, Schizophrenia

## Abstract

Although abnormal cortical gyrification has been consistently reported in patients with schizophrenia, whether gyrification abnormalities reflect a genetic risk for the disorder remains unknown. This study investigated differences in cortical gyrification between unaffected relatives (URs) with high genetic loading for schizophrenia and healthy controls (HCs) to identify potential genetic vulnerability markers. A total of 50 URs of schizophrenia patients and 50 matched HCs underwent T1-weighted magnetic resonance imaging to compare whole-brain gyrification using the local gyrification index (lGI). Then, the lGI clusters showing significant differences were compared between the UR subgroups based on the number of first-degree relatives with schizophrenia to identify the effect of genetic loading on cortical gyrification changes. The URs exhibited significantly lower cortical gyrification than the HCs in clusters including medial parieto-occipital and cingulate regions comprising the bilateral precuneus, cuneus, pericalcarine, lingual, isthmus cingulate, and posterior cingulate gyri. Moreover, URs who had two or more first-degree relatives with schizophrenia showed greater gyrification reductions in these clusters than those who had at least one first-degree relative with schizophrenia. Our findings of reduced gyrification in URs, which are consistent with accumulated evidence of hypogyria observed in regions showing patient-control differences in previous studies, highlight that such hypogyria in posteromedial regions may serve as a genetic vulnerability marker and reflect early neurodevelopmental abnormalities resulting from a genetic risk for schizophrenia.

## Introduction

Schizophrenia is highly heritable in that biological relatives of patients have an increased risk of developing the disease, and genetic factors constitute significant risk factors in schizophrenia^1^. Endophenotype strategies have been adopted in an effort to identify genetic underpinnings in the etiology of schizophrenia to allow early detection and prevention of the disease^2^. An endophenotype is a state-independent and measurable biological trait found in unaffected relatives (URs) of probands at a higher rate than in the general population. Abnormalities in gyrification, the developmental process of forming cortical sulcal and gyral patterns, have been proposed as a candidate structural endophenotype for schizophrenia^3^, and this hypothesis has been supported by its characteristics of high heritability^4^ and consistent observations in schizophrenia patients^5–9^. In particular, schizophrenia is widely accepted as a disease attributable to genetic factors that initiate abnormal neurodevelopmental processes before the onset of illness^10,11^. Given that the formation of cortical folding is largely determined during early neurodevelopment and strongly influenced by genetic factors^12–14^, investigating gyrification changes in URs may help identify a genetic vulnerability marker reflecting the early neurodevelopmental etiopathology of schizophrenia.

The local gyrification index (lGI) method, which considers the intrinsic three-dimensional morphology of the cortical surface, has enabled accurate examination of regionally specific gyral abnormalities in the whole-brain^15^. While several inconsistent results have been reported^16,17^, reduced gyrification was the main finding in most lGI studies^18^, and both patients with schizophrenia and patients with first-episode psychosis (FEP) showed common widespread hypogyria in the medial parieto-occipital and cingulate regions^5,6,8,9,19^. However, with respect to gyrification abnormalities reported in URs of schizophrenia patients^19–24^, the results have been conflicting and inconclusive, and only one existing lGI study reported a negative finding (Table 1). These inconsistent results may be mainly attributable to different subject characteristics and inclusion criteria for the relatives. The risk of developing schizophrenia increases proportionally with familial closeness and the number of affected probands^25^. However, previous studies have included URs with relatively low genetic loading for schizophrenia (i.e., with only one first-degree relative with schizophrenia), which might have reduced the probability of finding crucial cortical folding differences caused by genetic risk. Moreover, relatives of schizophrenia patients with different symptomatic and functional statuses have been included as ‘unaffected relatives of patients with schizophrenia’, complicating identification of the pure genetic effects of schizophrenia on gyrification differences that exclude the influence of other psychiatric symptoms. For instance, the Edinburgh High Risk Study reported that more than 40% of URs of schizophrenia patients have psychiatric symptoms^26^, suggesting the possibility of the inclusion of URs with prodromal symptoms in previous studies, which might have also influenced their general functioning. In particular, negative results may have been observed in an lGI study of first-degree relatives of psychotic disorder probands with schizophrenia, psychotic bipolar, and schizoaffective disorders because these first-degree relatives had relatively low genetic loading for schizophrenia and were recruited based on relationships with probands who had different psychotic illnesses^19^.

**Table 1:** Cross-sectional gyrification studies in unaffected relatives of patients with schizophrenia.

Author (year)	Participating subjects	Mean age (SD)	Gyrification analysis method	Significant results
Harris et al. (2004)^21^	16 HR-developed SCZ 14 HR-did not develop SCZ	20.1 (2.4) 21.8 (3.0)	Manual tracing 2-D GI	↑ right prefrontal lobe GI in HR who developed SCZ.
Harris et al. (2007)^22^	128 HR-developed SCZ 17 HR-did not develop SCZ	21.3 (3.0) 20.2 (2.5)	Automated 2-D GI	↑ right prefrontal lobe GI in HR who developed SCZ.
Vogeley et al. (2001)^24^	12 Siblings + SCZ or SZA 12 Unaffected HR	30.8 (7.3)	Manual tracing 2-D GI	↑ right prefrontal lobe GI in siblings with schizophrenia or schizoaffective disorder.
Jou et al. (2005)^23^	9 Unaffected HR 12 Controls	15.5 (2.9) 14.5 (5.3)	Manual tracing 2-D GI	↓ left hemisphere GI in HR.
Falkai et al. (2007)^20^	48 SCZ 29 Unaffected HR + psy. DX 53 Unaffected HR − psy. DX 41 Controls	41.4 (12.9) 44.8 (16.8) 34.6 (10.6) 32.5 (13.3)	Manual tracing 2-D GI	↑ frontal but not parieto-occipital GI in SCZ and unaffected HR. No difference between HR.
Nanda et al. (2014)^19^	157 SCZ 90 SZA 141 BP 300 First-degree relatives 243 Controls	34.3 (12.2) 35.7 (12.2) 36.6 (13.0) 39.8 (16.1) 37.5 (12.3)	Local gyrification index (lGI)	↓ bilateral caudal anterior cingulate, right posterior cingulate in probands. Nonsignificant ↓ in same regions in relatives with axis II cluster A personality disorders.

*SCZ* schizophrenia, *HR* high risk, *SZA* schizoaffective, *BP* psychotic bipolar, + with, − without; *psy*. *DX* psychiatric diagnosis, *2-D* 2-dimensional, *GI* gyrification index.

However, URs with axis II cluster A personality disorders exhibited reduced gyrification with a trend approaching statistical significance in regions of hypogyria evidenced in their probands^19^. As they were characterized by traits such as schizotypy, which is considered a genetic risk factor for schizophrenia, this result suggests that hypogyria in the cingulate may serve as a genetic vulnerability marker for schizophrenia. Furthermore, patients with 22q11 deletion syndrome (22q11DS), which reflects another major genetic liability for schizophrenia, also showed significantly decreased gyrification in a large medial cluster extending from the posterior cingulate to the precuneus regions, supporting the notion that hypogyria in the medial parietal lobe is possibly associated with a genetic risk for schizophrenia^15^. However, it remains unknown whether reduced gyrification represents an endophenotype specifically for schizophrenia. To our knowledge, there has been no direct investigation of whole-brain gyrification in asymptomatic URs with high genetic loading for schizophrenia, which could increase the probability of detecting pure effects of genetic risk for schizophrenia on cortical gyrification changes.

In this study, we compared cortical gyrification in asymptomatic, high-functioning URs with high genetic loading for schizophrenia and healthy controls (HCs) using the lGI to identify potential endophenotypes for schizophrenia. Based on previous lGI studies reporting reduced gyrification in regions of case-control differences in schizophrenia patients and their relatives^5,6,8,9,19^, we hypothesized that our URs of schizophrenia patients would exhibit hypogyrification patterns in the medial parieto-occipital and cingulate regions compared to HCs as a genetic vulnerability marker. Furthermore, to strengthen the influence of genetic liability on reduced cortical folding, URs were divided into two subgroups according to the number of first-degree relatives with schizophrenia; in this exploratory analysis, we assessed whether URs with higher genetic loading for schizophrenia showed greater hypogyria in the lGI clusters with significant differences between URs and HCs.

## Results

### Subject background

The demographic and clinical backgrounds of the groups are presented in Table 2. There were no significant group differences between the URs and HCs. For exploratory analysis, the URs were divided into the following two groups based on the number of affected first-degree relatives as described in previous studies^27,28^: (1) the “multiplex” group included URs who had at least two first-degree relatives or monozygotic twins with schizophrenia (*n* = 9) and (2) the “simplex” group included URs who had only one first-degree relative with schizophrenia and at least one other affected second- to third-degree relative (*n* = 41). No significant group differences were found between the multiplex and simplex UR subgroups except for the sex ratio (Z = 5.767; *p* = 0.025).

**Table 2:** Demographic information and clinical variables of the groups.

	URs (*n* = 50)	HCs (*n* = 50)	*T* or *X*^*2*^	*p*
Age (years)	22.9 ± 5.3	22.2 ± 4.6	0.766	0.446
Sex (male/female)	29/21	30/20	0.041	1.000
Handedness (right/left)	47/3	45/5	0.543	0.715
IQ	107.2 ± 13.3	105.3 ± 8.6	0.875	0.384
Education (years)	13.7 ± 2.6	13.8 ± 1.8	−0.135	0.893
SIPS total	4.1 ± 4.0			
SIPS positive	1.0 ± 1.5			
SIPS negative	1.5 ± 1.7			
SIPS disorganized	0.3 ± 0.6			
SIPS general	1.3 ± 1.6			
BPRS	26.7 ± 3.4			
HAMA	2.3 ± 2.5			
HAMD	2.6 ± 3.3			
GAF	83.4 ± 8.0			
ICV (cm^3^)	1551.6 ± 158.0	1567.9 ± 163.1	−0.508	0.612

	Multiplex (*n* = 9)	Simplex (*n* = 41)	*Z* or *X*^*2*^	*p*
Age (years)	24.0 ± 4.3	22.7 ± 5.5	−0.824	0.426
Sex (male/female)	2/7	27/14	5.767	0.025*
Handedness (right/left)	9/0	38/3	0.701	0.623
IQ	103.0 ± 11.3	108.2 ± 13.6	−1.113	0.272
Education (years)	14.1 ± 1.5	13.7 ± 2.8	−0.587	0.568
SIPS total	4.1 ± 4.3	4.1 ± 4.0	−0.267	0.804
SIPS positive	0.2 ± 0.4	1.1 ± 1.7	−1.515	0.184
SIPS negative	1.9 ± 1.8	1.4 ± 1.6	−0.981	0.356
SIPS disorganized	0.3 ± 0.5	0.3 ± 0.6	−0.142	0.921
SIPS general	1.7 ± 2.7	1.3 ± 1.3	−0.724	0.502
BPRS	27.3 ± 4.2	26.6 ± 3.2	−0.283	0.785
HAMA	2.4 ± 2.4	2.3 ± 2.6	−0.193	0.862
HAMD	2.9 ± 4.4	2.5 ± 3.1	−0.516	0.619
GAF	84.9 ± 9.3	83.0 ± 7.8	−1.185	0.250
ICV (cm^3^)	1455.0 ± 150.0	1572.8 ± 153.3	−1.793	0.073

*URs* unaffected relatives, *HCs* healthy controls, *IQ* intelligent quotient, *SIPS* Structured Interview for Prodromal Syndromes, *BPRS* Brief Psychiatric Rating Scale, *HAMA* Hamilton Rating Scale for Anxiety, *HAMD* Hamilton Rating Scale for Depression, *GAF* Global Assessment of Functioning, *ICV* intracranial volume.

*Note:* Data are presented as the mean ± S.D. *p < 0.05.

### Group comparisons of cortical gyrification

**Fig. 1:**
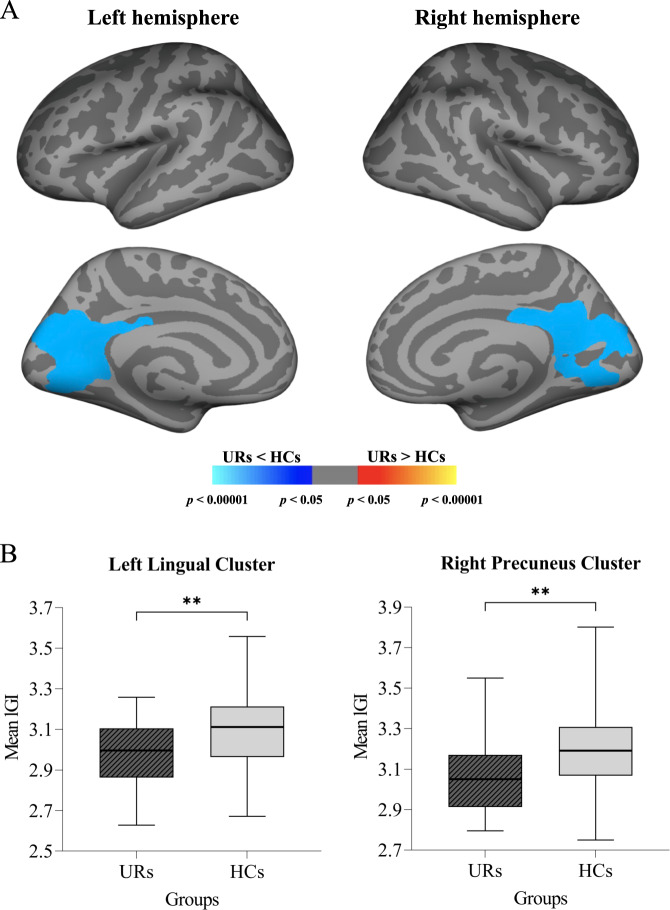
Group differences in the local gyrification index (lGI) between the unaffected relatives (URs) and healthy controls (HCs). **A** Statistical maps of the left and right hemispheres are shown in the lateral and medial views, respectively. The maps are shown for the clusters with significantly reduced lGI in the URs after clusterwise correction for multiple comparisons (*p* < 0.05). **B** Bar graph of mean lGI values extracted from the left lingual cluster and right precuneus cluster showing significant group differences. The error bars indicate 95% confidence intervals. **p < 0.01.

The results of the whole-brain lGI analysis are summarized in Table 3. Significantly reduced gyrification was observed in the URs of patients with schizophrenia compared to the HCs across several regions of the cortex, including one cluster in the left hemisphere and one cluster in the right hemisphere. The left cluster showing a significantly reduced lGI (*p* < 0.001) had a peak vertex located within the lingual gyrus covering portions of the precuneus, cuneus, pericalcarine, lingual, isthmus cingulate, parahippocampal, and posterior cingulate gyri. The right cluster showing a significantly reduced lGI (*p* < 0.001) had a peak vertex within the precuneus gyrus comprising portions of the precuneus, cuneus, pericalcarine, lingual, isthmus cingulate, and posterior cingulate gyri. No region had a significantly increased lGI in the URs compared to the HCs (Fig. 1A).

**Table 3:** Description of clusters with significantly reduced local gyrification index (lGI) in the unaffected relatives (URs) compared to the healthy controls (HCs) in the left and right hemispheres after clusterwise correction for multiple comparisons using Monte Carlo simulation (*p* < 0.05).

Cluster number	Peak vertex cluster	VtxMax	Size (mm^2^)	Peak vertex MNI			CWP (*p*)
				x	y	z	
1	L lingual	44064	5077.45	−16.6	−72.0	−4.6	0.00010***
2	R precuneus	146997	4597.33	5.4	−56.4	30.0	0.00010***

*L* left hemisphere, *R* right hemisphere, *VtxMax* number of peak vertices in the significant cluster, *MNI* Montreal Neurological Institute (coordinate system), *CWP* clusterwise probability and the nominal *p* value, ***p < 0.001.

Additionally, significantly reduced mean lGI values in the left lingual cluster (*t* = −3.296; *p* = 0.001; Cohen’s *d* = 0.659) and right precuneus cluster (*t* = −2.745; *p* = 0.007; Cohen’s *d* = 0.549) were demonstrated in the URs compared to the HCs. The results are shown as box plots in Fig. 1B.

### Effect of genetic loading on cortical gyrification

**Fig. 2:**
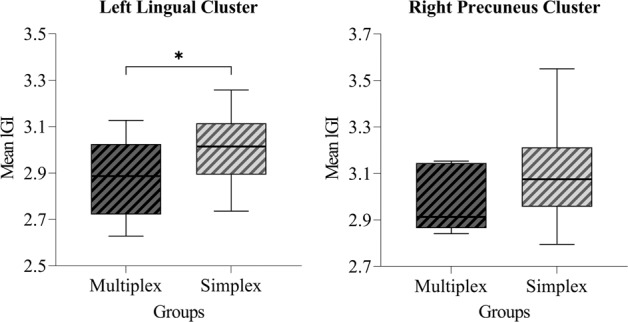
Relationships between genetic loading and mean local gyrification index (lGI) values within the clusters showing significantly reduced gyrification in the unaffected relatives (URs). The comparison between the two subgroups of URs in terms of the extracted mean lGI values from the clusters showing significant differences between the URs and healthy controls (HCs). The error bars indicate 95% confidence intervals. *p < 0.05.

To demonstrate the effect of genetic loading on reduced gyrification in the URs as an exploratory analysis, the comparison between the two subgroups of URs in terms of the extracted mean lGI values from the clusters showing significant differences between the URs and HCs was conducted. Compared to the simplex group, the multiplex group demonstrated a significantly reduced mean lGI in the left lingual cluster (Z = −2.058; *p* = 0.039; effect size *r* = −0.291). The right precuneus cluster also showed a tendency toward decreased gyrification in the multiplex group (Z = −1.654; *p* = 0.101; effect size *r* = −0.234), although the difference was not statistically significant. The results are shown as box plots in Fig. 2.

## Discussion

To our knowledge, this is the first MRI study reporting significant differences in whole-brain gyrification between asymptomatic, high-functioning URs who have high genetic loading for schizophrenia and HCs. The present study revealed significantly reduced cortical folding in the medial parieto-occipital and cingulate regions, which is consistent with previously reported findings in patients with schizophrenia, FEP, and other psychotic illnesses^5,6,8,9,19^. Furthermore, the URs with higher genetic loading who had at least two first-degree relatives with schizophrenia showed significantly greater cortical gyrification reductions than those who had one first-degree relative and at least one second- to third-degree relative with schizophrenia, supporting the effect of the level of genetic loading for schizophrenia on the degree of hypogyrification. Our findings confirm the accumulated evidence of significantly reduced gyrification in regions with schizophrenia patient-control differences from previous studies and further highlight that such hypogyria in posteromedial regions may serve as a stable structural endophenotype reflecting an early neurodevelopmental deficit resulting from a genetic risk for schizophrenia.

Our UR groups, along with patients with schizophrenia and FEP in previous studies^5,6,8,9,29,30^, showed significant gyral anomalies on the bilateral medial surface of the hemisphere specifically in the precuneus and posterior cingulate extending to the lingual gyrus. Since the recruited URs in this study were asymptomatic and had high genetic loading for schizophrenia, the hypogyria in these regions may be attributed to genetic vulnerability rather than the results of illness progression or medication effects. In fact, major genetic risk factors for schizophrenia seem to contribute to the cortical folding reduction in that patients with 22q11DS demonstrated significant hypogyrification patterns in the large medial cluster of the parietal lobe compared to controls^15^. Moreover, URs with axis II cluster A personality disorders, a psychopathology trait reflecting genetic liability to schizophrenia, also exhibited reduced gyrification in cingulate regions, with a trend approaching statistical significance^19^, supporting the hypothesis that hypogyria in the medial parieto-occipital and cingulate regions may serve as a stable genetic markers of vulnerability to schizophrenia. Particularly, hypogyria in these regions was associated with the persistence of psychotic symptom burden that remained regardless of antipsychotic treatment, as demonstrated by the nonresponders among patients with FEP^9^. A previous study in schizophrenia patients with medication-resistant auditory hallucinations also reported hypogyria in the posterior cingulate sulcus and anterior calcarine fissure as a trait feature of vulnerability to hallucinations^29^.

Notably, our URs with two or more first-degree relatives with schizophrenia (i.e., multiplex) showed greater hypogyrification patterns in the medial parieto-occipital and cingulate regions than those with one first-degree relative and at least one second- to third-degree relative with schizophrenia (i.e., simplex). It has been hypothesized that the genetic risk for schizophrenia increases proportionally with familial closeness and the number of affected probands^25,28^. Supporting this hypothesis, previous studies have reported significant relationships between the level of genetic liability for schizophrenia and the degree of cortical structural changes, including cortical thinning^31^ and gray matter volume reduction^32^ in the cingulate regions. Furthermore, the multiplex group showed a trend toward greater reductions in default mode network (DMN) connectivity in the precuneus and anterior cingulate cortex (ACC) than the simplex group, suggesting that dysfunction in these regions is related to genetic risk for schizophrenia^27^. Consistently, our findings demonstrated the effects of genetic loading for schizophrenia on the amount of reduced cortical folding in the posteromedial cortex, which supports the notion that the precuneus/posterior cingulate regions reflect one of the most genetically vulnerable regions for schizophrenia.

Abnormal gyrification has been repeatedly suggested as a candidate endophenotype for schizophrenia^3,19^. However, previous studies of URs of schizophrenia patients have reported conflicting results regarding gyrification patterns, mainly due to the heterogeneous characteristics of URs and different methods used to measure gyrification. In particular, the previous work by Nanda et al. (2014), one of the most prominent lGI studies in this line, examined gyrification in first-degree relatives of patients with different psychotic disorders. However, they treated the relatives of patients with schizophrenia, schizoaffective disorder, and psychotic bipolar disorder as an overall group in the same sample to examine whether gyrification qualifies as an endophenotype broadly marking psychosis liability. Furthermore, this group may have included relatives with relatively low genetic loading for schizophrenia (i.e., only one first-degree relative with schizophrenia), which might also have reduced the probability of finding significant gyrification differences caused by genetic risk for schizophrenia, and thereby resulted in a negative finding. To investigate whether reduced gyrification represents an endophenotype specifically for schizophrenia, URs with high genetic loading for schizophrenia were recruited in our study by including URs who had at least one first-degree relative with schizophrenia and one or more other affected first- to third-degree relatives. In addition, some of the URs in previous studies may have had certain psychiatric symptoms or prodromal syndromes that might also have influenced their general functioning, making it difficult to identify a pure effect of genetic risk for schizophrenia on changes in cortical folding. Compared to these previous studies, our findings were further bolstered by applying strict inclusion criteria for subjects in that URs having prodromal symptoms were screened out by measuring Structured Interview for Prodromal Syndromes (SIPS) scores, and subjects who had any general psychopathology were excluded by assessing Brief Psychiatric Rating Scale (BPRS), Hamilton Rating Scale for Anxiety (HAMA), and Hamilton Rating Scale for Depression (HAMD) scores to ensure they were all asymptomatic; Global Assessment of Functioning (GAF) scores were also evaluated to confirm that all URs were well functioning. Taken together, these analyses allowed us to investigate the pure effect of shared genetic factors of schizophrenia on cortical gyrification changes as a biological trait that was free from the aforementioned confounding factors, which is consistently evidenced in other genetic models for schizophrenia^15,19^.

Schizophrenia is widely accepted as a neurodevelopmental disease in that deficits of the brain are determined by genetic factors early in life but are not fully established until adolescence^10,11^. Longitudinal studies of subjects at genetic high risk have indicated that progressive brain structural alterations predate the onset of psychosis^33^, suggesting that genetic vulnerability to schizophrenia might affect cortical changes during neurodevelopment. In fact, prenatal genetic insults are known to influence embryonic and early neurodevelopmental processes, such as neuronal proliferation and differentiation, synaptic formation, and myelination, leading to prolonged alterations in cortical structure and connectivity in schizophrenia^34^. As the formation of gyral and sulcal patterns occurs mainly in early neurodevelopment and is strongly influenced by genetic factors during this period^12,13^, investigating gyrification differences in asymptomatic URs with high genetic loading may help reveal the effects of genetic risk on the neurodevelopmental etiopathology of schizophrenia. Because cortical gyrification mainly increases before and shortly after birth and undergoes minor decreases until becoming relatively stable in early childhood^12,14^, reduced gyrification in URs relative to HCs may result from deficits in an earlier phase of increasing cortical maturation or an excessive decrease in later neurodevelopmental processes. Furthermore, the alterations in gyrification may reflect underlying deficits in white matter integrity during cortical maturation^35–37^, which are known to be disrupted in both schizophrenia patients and their URs^38,39^.

There are several limitations of the present study that should be considered. The cross-sectional nature of the study makes it difficult to investigate whether abnormal cortical folding increases the possibility of developing schizophrenia. Thus, longitudinal MRI analyses in URs of patients with schizophrenia would be required to identify alterations in neurodevelopmental trajectories and confirm the role of gyrification deficits as a vulnerability marker for schizophrenia. Moreover, the sample size for the multiplex UR subgroup was relatively small. Thus, our findings of the effect of the level of genetic loading for schizophrenia on the degree of hypogyria should be considered exploratory and further tested in a larger cohort of both simplex and multiplex UR subgroups.

In conclusion, our findings of asymptomatic, high-functioning URs with high genetic loading for schizophrenia demonstrate that genetic vulnerability for schizophrenia is characterized by reduced gyrification, and, in particular, the posteromedial regions may serve as the most vulnerable regions to genetic liability in schizophrenia. Taken together with previous findings in patients with schizophrenia, our findings confirm that such hypogyria may represent a trait characteristic of schizophrenia and reflect early neurodevelopmental disturbances resulting from a genetic risk for schizophrenia. Our findings of the relationship between higher genetic loading for schizophrenia and greater cortical gyrification reduction further support the possibility of hypogyria as a candidate structural endophenotype that may contribute to the understanding of the genetic underpinnings of a neurobiological etiology in schizophrenia. Future investigations of genes involved in the neurodevelopmental mechanisms of cortical gyrification associated with increased risk for developing schizophrenia will identify the genetic influence on the neurodevelopmental etiopathology of schizophrenia.

## Methods

### Participants

Fifty URs of schizophrenia patients were recruited from the Seoul Youth Clinic^40^, a center for prospective, longitudinal investigation of people at high risk for schizophrenia, and from the inpatient and outpatient clinics of the Department of Psychiatry at Seoul National University Hospital. The URs were defined as asymptomatic subjects with high genetic risk who had at least one first-degree relative with schizophrenia and one or more other affected first- to third-degree relatives. This standard has the benefit of including URs with high genetic loading and excluding relatives of patients with sporadic schizophrenia. The probands of URs were assessed for schizophrenia according to the *Diagnostic and Statistical Manual of Mental Disorders*, fourth edition (DSM-IV) by trained psychiatrists. The family history of psychiatric disorders and the degree of genetic loading for schizophrenia were evaluated in URs using the Family Interview for Genetic Studies^41^. To assess clinical status upon admission into the study, the Structured Interview for Prodromal Syndromes (SIPS)^42^ was used to screen for prodromal psychotic symptoms. URs with SIPS scores exceeding certain criteria for clinical high risk (CHR) for psychosis (any P1–P5 ≥ 3) were excluded from this study. The URs were also administered the Brief Psychiatric Rating Scale (BPRS) to measure general psychopathologic symptom severity and the Hamilton Rating Scale for Depression (HAMD)^43^ and the Hamilton Rating Scale for Anxiety (HAMA)^44^ to assess the severity of depression and anxiety, respectively. In addition, the Global Assessment of Functioning (GAF) scale was also assessed to evaluate the overall functioning of subjects.

Fifty HCs matched for age, sex, handedness, intelligence quotient (IQ), and years of education were recruited via internet advertisements and screened for the presence of psychiatric disorders or symptoms using the Structured Clinical Interview for DSM-IV–Non-Patient Version (SCID-NP). Potential HCs were excluded if they reported past or current axis-I diagnoses and had any first- to third-degree biological relatives with a lifetime history of any major psychiatric disorders.

The IQ of all participants was measured with the Korean version of the Wechsler Adult Intelligence Scale (K-WAIS) to estimate intelligence. The exclusion criteria for all participants included a lifetime diagnosis of a psychotic disorder, substance abuse or dependence, neurological disease or head injury, evidence of any other mental illness, or intellectual disability (IQ < 70). The present study was also approved by the Institutional Review Board at Seoul National University Hospital, and written informed consent was obtained from all subjects and from the parents of subjects under 18 years of age after the procedures had been fully explained.

### Image acquisition

The subjects were scanned, and T1-weighted (T1) images were acquired using a Siemens 3T MAGNETOM Trio MR scanner (Siemens, Erlangen, Germany). For the T1 images, a three-dimensional (3D) magnetization-prepared rapid acquisition gradient-echo (MPRAGE) sequence and the following imaging parameters were used: repetition time, 1670 ms; echo time (TE), 1.89 ms; voxel size, 1.0 × 0.98 × 0.98 mm^3^; field of view, 250 mm; flip angle, 9°; and 208 slices. To ensure quality control, the acquired MRI images were visually inspected for any artifacts or malformation of brain structures.

### Image processing

The preprocessing of T1 images was performed using FreeSurfer version 5.3.0 (http://surfer.nmr.mgh.harvard.edu/) according to the standard and automatic reconstruction algorithm^45^. This processing stream consists of automated transformation to Talairach space, normalization of intensity, removal of nonbrain tissue, and segmentation of gray/white matter tissue resulting in a white mesh and a pial mesh composed of approximately 150,000 vertices for each hemisphere.

Gyrification of the entire cortex was computed at each vertex using the lGI. This three-dimensional approach of assessing gyrification is a vertexwise extension of the classic two-dimensional GI approach, which represents the ratio of the total pial surface over the outer perimeter on coronal sections^46^. The lGI method considers the inherent three-dimensional nature of the cortex, including buried sulci, neither restricted by sulcal walls nor biased by the orientation or thickness of the slices. It measures the amount of cortex buried within the sulcal folds compared with the amount of visible cortex across the whole-brain cortical surface, allowing regional specific localization of gyral abnormalities^15^.

Briefly, the automatic lGI computation involved the creation of an outer smoothed surface tightly wrapping the pial surface and estimation of 800 overlapping 25-mm spherical three-dimensional regions of interest (ROIs) on the smoothed outer surface and of their corresponding paired circular ROIs on the pial surface. The lGI value at each vertex of the cortical surface was computed as the ratio between the smoothed outer surface and buried cortex resulting in the measurement of individual maps.

### Statistical analysis

Data were analyzed using Statistical Package for Social Sciences (IBM SPSS Version 22). The demographic and clinical characteristics between URs and HCs were examined with independent t-tests or χ2 tests. A whole-brain lGI comparison between groups was performed with FreeSurfer. The lGI values were mapped on the average template subject (fsaverage) for each subject to perform the contrasts of the vertexwise analysis in Query Design Estimate Contrast (QDEC). A general linear model (GLM) for surface-based group analyses was conducted to assess regional group differences in lGI at each vertex for the right and left hemispheres separately while controlling for age and sex. A smoothing Gaussian kernel of 5-mm full-width at half-maximum (FWHM) was applied to the maps. The significant cluster-forming threshold was set to *p* < 0.05, and clusters were corrected for multiple comparisons by Monte Carlo simulation. For clusters showing significant group differences, mean lGI values were extracted for each participant, and independent t-tests were performed to compare gyrification patterns between the groups.

To further investigate the effect of genetic loading on clusters showing significant differences between the URs and HCs, the URs were divided into two subgroups based on the number of their first-degree relatives with schizophrenia. The demographic and clinical variables and the mean lGI values were compared between the two subgroups of URs using nonparametric Mann-Whitney U tests or χ2 tests due to the small sample size.

### Reporting summary

Further information on research design is available in the Nature Research Reporting Summary linked to this article.

## Supplementary information

Reporting Summary

## Data Availability

The data that support the results of this study are available from the corresponding author upon reasonable request. The data are not publicly available because they contain information that might compromise the privacy of the research participants.

## Code availability

The code for data processing and statistical analysis in this study are available from the corresponding author upon reasonable request.
